# 2-Acetamido-2-de­oxy-3-*O*-β-d-galactopyranosyl-d-glucose dihydrate

**DOI:** 10.1107/S1600536809024775

**Published:** 2009-07-04

**Authors:** Masahisa Wada, Kayoko Kobayashi, Mamoru Nishimoto, Motomitsu Kitaoka, Keiichi Noguchi

**Affiliations:** a Department of Biomaterials Science, Graduate School of Agricultural and Life Science, The University of Tokyo, Yayoi 1-1-1, Bunkyo-ku, Tokyo 113-8657, Japan; b National Food Research Institute, National Agriculture and Food Research Organization, 2-1-12 Kanondai, Tsukuba, Ibaraki 305-8642, Japan; c Instrumentaion Analysis Center, Tokyo University of Agriculture & Technology, 2-24-16 Naka-cho, Koganei, Tokyo 184-8588, Japan

## Abstract

In the title compound, C_14_H_25_NO_11_·2H_2_O, the primary hydroxyl group connected to the anomeric C atom of the *N*-acetyl-β-d-glucopyran­ose residue exhibits positional disorder, with occupancy factors for the α and β anomers of 0.77 and 0.23, respectively. The two torsion angles (Φ and Ψ) and the bridge angle (τ) that describe conformation of the glycosidic linkage between the galactopyran­ose and glucopyran­ose rings are Φ = −81.6 (3)°, Ψ = 118.1 (2)° and τ = 115.2 (2)°. Two water mol­ecules stabilize the mol­ecular packing by forming hydrogen bonds with the saccharide residues.

## Related literature

For the synthesis of the title compound, see: Kitaoka *et al.* (2005 ▶); Nishimoto & Kitaoka (2007*a* ▶,*b* ▶). For the conformation of saccharide rings, see: Cremer & Pople (1975 ▶).
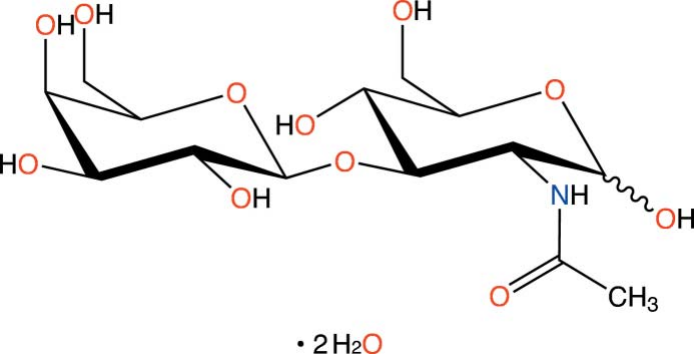


## Experimental

### 

#### Crystal data

C_14_H_25_NO_11_·2H_2_O
*M* *_r_* = 419.38
Orthorhombic, 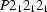

*a* = 8.284 (1) Å
*b* = 12.841 (1) Å
*c* = 17.503 (1) Å
*V* = 1861.9 (3) Å^3^
*Z* = 4
Synchrotron radiation
λ = 0.80000 Å
μ = 0.13 mm^−1^
*T* = 95 K
0.10 × 0.10 × 0.10 mm

#### Data collection

ADSC Quantum 210r diffractometer
Absorption correction: none
25787 measured reflections
2153 independent reflections
2046 reflections with *I* > 2σ(*I*)
*R*_int_ = 0.047

#### Refinement

*R*[*F* ^2^ > 2σ(*F* ^2^)] = 0.042
*wR*(*F* ^2^) = 0.115
*S* = 1.06
2153 reflections
264 parameters
H-atom parameters constrained
Δρ_max_ = 0.27 e Å^−3^
Δρ_min_ = −0.31 e Å^−3^

### 

Data collection: *UGUI* (Structural Biology Research Center, 2005 ▶); cell refinement: *HKL-2000* (Otwinowski & Minor, 1997 ▶); data reduction: *HKL-2000*; program(s) used to solve structure: *SHELXS86* (Sheldrick, 2008 ▶); program(s) used to refine structure: *SHELXL97* (Sheldrick, 2008 ▶); molecular graphics: *ORTEPIII* (Burnett & Johnson, 1996 ▶); software used to prepare material for publication: *SHELXL97*.

## Supplementary Material

Crystal structure: contains datablocks global, I. DOI: 10.1107/S1600536809024775/is2433sup1.cif

Structure factors: contains datablocks I. DOI: 10.1107/S1600536809024775/is2433Isup2.hkl

Additional supplementary materials:  crystallographic information; 3D view; checkCIF report

## Figures and Tables

**Table:** 

C1—O1—C9	115.2 (2)

**Table:** 

O5—C1—O1—C9	−81.6 (3)
C2—C1—O1—C9	159.0 (2)
C1—O1—C9—C10	118.0 (2)
C1—O1—C9—C8	−123.3 (2)

**Table 2:** Hydrogen-bond geometry (Å, °)

*D*—H⋯*A*	*D*—H	H⋯*A*	*D*⋯*A*	*D*—H⋯*A*
N1—H1*N*⋯O2*W*^i^	0.88	2.05	2.923 (3)	169
O2—H2*O*⋯O12^ii^	0.85	1.80	2.642 (3)	170
O3—H3*O*⋯O2*W*	0.85	1.86	2.702 (3)	169
O4—H4*O*⋯O13^ii^	0.93	1.95	2.803 (3)	150
O6—H6*O*⋯O1*W*^iii^	0.85	1.79	2.624 (3)	168
O10—H10*O*⋯O6^iv^	0.96	1.81	2.705 (3)	154
O1*W*—H11*W*⋯O10^iv^	0.85	1.85	2.696 (3)	175
O12—H12*O*⋯O13^v^	0.85	1.96	2.784 (3)	162
O1*W*—H12*W*⋯O4	0.87	1.90	2.759 (3)	173
O2*W*—H21*W*⋯O11^vi^	0.90	1.94	2.772 (3)	154
O2*W*—H22*W*⋯O6^vii^	0.85	1.91	2.757 (3)	171
O71—H71*O*⋯O2^i^	0.86	1.87	2.683 (3)	159
O72—H72*O*⋯O1*W*^v^	0.84	2.04	2.545 (9)	119

Symmetry codes: (i) 
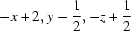
; (ii) 
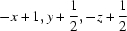
; (iii) 
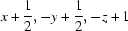
; (iv) 
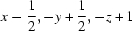
; (v) 
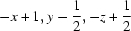
; (vi) 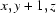; (vii) 
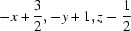
.

**Fig. 1.**
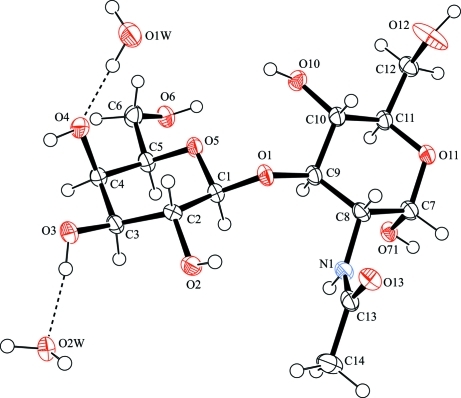
Displacement ellipsoid plot and atomic numbering scheme of compound (I). The ellipsoids are drawn at the 50% probability level, and the H atoms are shown as small spheres with arbitrary radii. Broken lines indicate hydrogen bonds. The minor conformer of the disordered part has been omitted for clarity.

## supplementary crystallographic information

### Comment

It is widely accepted that oligosaccharides other than lactose in human milk (human milk oligosaccharides, HMOs) play a key role in the growth of *Bifidobacteria* in the gut. *Bifidobacteria*, Gram-positive anaerobes, are considered to be beneficial for human health. Recently, a unique metabolic pathway specific for lacto-N-biose I (Gal-β1→3GlcNAc, LNB) was found using *Bifidobacteria* (Kitaoka *et al.*, 2005; Nishimoto & Kitaoka, 2007*a*). LNB is one of the basic core disaccharides of HMOs and is suggested to be a bifidus factor.

The molecular structure of compound (I) is shown in Fig. 1. There are two water molecules per LNB molecule in the crystal lattice. The primary hydroxyl group connected to the anomeric carbon atom of the GlcNAc residue exhibits disorder, with occupancy factors of O71 (α anomer) and O72 (β anomer) of 0.77 and 0.23, respectively.

The Gal ring is close to the ideal ^4^*C*_1_ chair conformation with ring puckering parameters (Cremer & Pople, 1975) of *Q* = 0.556 (3) Å, Θ = 7.1 (3)° and Φ = 353 (2)° for the atom sequence O5—C1—C2—C3—C4—C5. The other GlcNAc ring is also close to the ideal chair conformation, with *Q* = 0.618 (3) Å, Θ = 3.8 (3)°, and Φ = 198 (4)° for the atom sequence O11—C7—C8—C9—C10—C11.

The conformation about the linkage between the Gal and GlcNAc rings is characterized by the torsion angles of Φ (O5—C1—O1—C9) and Ψ (C1—O1—C9—C10), and the bridge angle τ (C1—O1—C9). The values obtained in this study are Φ = -81.6 (3)°, Ψ = 118.1 (2)° and τ = 115.2 (2)° (Table 1).

The conformation of the hydroxymethyl group is defined by two sets of torsion angle: χ and χ'. The values for the Gal ring were χ (O5—C5—C6—O6) = 79.5 (3)° and χ' (C4—C5—C6—O6) = -157.9 (2)°, indicating values close to the *gt* conformation. The values for the GlcNAc ring are χ (O11—C11—C12—O12) = -64.1 (3)° and χ' (C10—C11—C12—O12) = 57.9 (3)°, indicating the *gg* conformation.

Both saccharide rings lie approximately parallel to the *bc* plane and the intermolecular hydrogen bonds were only along the *a*-axis (Table 2). Two water molecules stabilize the molecular packing by forming hydrogen bonds with sugar molecules in three dimensions.

### Experimental

Compound (I) was synthesized from sucrose and GlcNAc by the concurrent action of four enzymes: sucrose phosphorylase, UDP-glucose-hexose-1-phosphate uridylyltransferase, UDP-glucose 4-epimerase, and lacto-N-biose phosphorylase (Nishimoto & Kitaoka, 2007*b*). Single crystals suitable for X-ray analysis were obtained by slow diffusion of ethanol into an aqueous solution.

### Refinement

The anomalous scattering signal of (I) is too weak to predict the accurate absolute structure. Therefore, the merging of Friedel pair data was performed before the final refinement. The hydroxyl H atoms in the saccharides and water molecules, except for H72O, were located in a difference Fourier map. The H72O atom was positioned using the HFIX 83 instruction in the *SHELXL97* software package, with O—H = 0.84 Å. These hydroxyl H atoms were subsequently refined as a riding model, with *U*_iso_(H) = 1.2*U*_eq_(O). The methine, methylene, methyl and amide H atoms were positioned using the HFIX 13, HFIX 23, HFIX 137 and HFIX 43 instructions, with C—H = 1.00, 0.99, 0.98 and 0.88 Å, respectively. These C- and N-bound H atoms were also refined as a riding model, with *U*_iso_(H) = 1.2*U*_eq_(C) for the methine, methylene and amide H atoms, and *U*_iso_(H) = 1.5*U*_eq_(C) for methyl H atoms.

### Crystal data

**Table:**

C_14_H_25_NO_11_·2H_2_O	*F*(000) = 896
*M**_r_* = 419.38	*D*_x_ = 1.496 Mg m^−^^3^
Orthorhombic, *P*2_1_2_1_2_1_	Synchrotron radiation, λ = 0.80000 Å
Hall symbol: P 2ac 2ab	Cell parameters from 25787 reflections
*a* = 8.284 (1) Å	θ = 2.2–30.0°
*b* = 12.841 (1) Å	µ = 0.13 mm^−^^1^
*c* = 17.503 (1) Å	*T* = 95 K
*V* = 1861.9 (3) Å^3^	Block, colorless
*Z* = 4	0.10 × 0.10 × 0.10 mm

### Data collection

**Table:**

ADSC Quantum 210r diffractometer	2046 reflections with *I* > 2σ(*I*)
Radiation source: Photon Facrory NW12A	*R*_int_ = 0.047
silicon	θ_max_ = 30.0°, θ_min_ = 2.2°
Detector resolution: 9.7466 pixels mm^-1^	*h* = −10→10
ω scans	*k* = −16→16
25787 measured reflections	*l* = −21→21
2153 independent reflections	

### Refinement

**Table:**

Refinement on *F*^2^	Secondary atom site location: difference Fourier map
Least-squares matrix: full	Hydrogen site location: difmap&geom
*R*[*F*^2^ > 2σ(*F*^2^)] = 0.042	H-atom parameters constrained
*wR*(*F*^2^) = 0.115	*w* = 1/[σ^2^(*F*_o_^2^) + (0.0806*P*)^2^ + 0.7215*P*] where *P* = (*F*_o_^2^ + 2*F*_c_^2^)/3
*S* = 1.06	(Δ/σ)_max_ = 0.001
2153 reflections	Δρ_max_ = 0.27 e Å^−^^3^
264 parameters	Δρ_min_ = −0.31 e Å^−^^3^
0 restraints	Extinction correction: *SHELXL97* (Sheldrick, 2008), Fc^*^=kFc[1+0.001xFc^2^λ^3^/sin(2θ)]^-1/4^
Primary atom site location: structure-invariant direct methods	Extinction coefficient: 0.084 (6)

### Special details

**Table:**

Geometry. All e.s.d.'s (except the e.s.d. in the dihedral angle between two l.s. planes) are estimated using the full covariance matrix. The cell e.s.d.'s are taken into account individually in the estimation of e.s.d.'s in distances, angles and torsion angles; correlations between e.s.d.'s in cell parameters are only used when they are defined by crystal symmetry. An approximate (isotropic) treatment of cell e.s.d.'s is used for estimating e.s.d.'s involving l.s. planes.
Refinement. Refinement of *F*^2^ against ALL reflections. The weighted *R*-factor *wR* and goodness of fit *S* are based on *F*^2^, conventional *R*-factors *R* are based on *F*, with *F* set to zero for negative *F*^2^. The threshold expression of *F*^2^ > σ(*F*^2^) is used only for calculating *R*-factors(gt) *etc*. and is not relevant to the choice of reflections for refinement. *R*-factors based on *F*^2^ are statistically about twice as large as those based on *F*, and *R*- factors based on ALL data will be even larger.

### Fractional atomic coordinates and isotropic or equivalent isotropic displacement parameters (Å^2^)

**Table:**

	*x*	*y*	*z*	*U*_iso_*/*U*_eq_	Occ. (<1)
C1	0.7021 (3)	0.32502 (19)	0.34879 (14)	0.0205 (5)	
H1	0.8227	0.3214	0.3471	0.025*	
C2	0.6435 (3)	0.4253 (2)	0.31127 (15)	0.0211 (5)	
H2	0.5234	0.4232	0.3059	0.025*	
C3	0.6921 (3)	0.52070 (19)	0.35853 (15)	0.0215 (5)	
H3	0.8113	0.5310	0.3535	0.026*	
C4	0.6518 (3)	0.5082 (2)	0.44263 (16)	0.0226 (6)	
H4	0.7071	0.5646	0.4721	0.027*	
C5	0.7119 (4)	0.4031 (2)	0.47122 (15)	0.0235 (6)	
H5	0.8323	0.4017	0.4671	0.028*	
C6	0.6658 (4)	0.3832 (2)	0.55375 (16)	0.0279 (6)	
H61	0.6722	0.4494	0.5826	0.033*	
H62	0.5526	0.3585	0.5559	0.033*	
O1	0.6358 (2)	0.24111 (13)	0.30922 (11)	0.0217 (4)	
O2	0.7150 (2)	0.43673 (14)	0.23805 (11)	0.0241 (4)	
H2O	0.6648	0.4055	0.2024	0.029*	
O3	0.6135 (3)	0.61167 (14)	0.33229 (11)	0.0261 (5)	
H3O	0.6709	0.6420	0.2988	0.031*	
O4	0.4818 (3)	0.51573 (15)	0.45519 (11)	0.0268 (5)	
H4O	0.4508	0.5788	0.4331	0.032*	
O5	0.6475 (2)	0.31996 (13)	0.42597 (10)	0.0219 (4)	
O6	0.7678 (3)	0.30802 (14)	0.58916 (11)	0.0266 (5)	
H6O	0.7491	0.2462	0.5742	0.032*	
C7	0.8597 (3)	−0.00264 (19)	0.25298 (15)	0.0220 (5)	
H71	0.8919	−0.0327	0.2025	0.026*	0.77
H72	0.9533	0.0120	0.2874	0.026*	0.23
C8	0.7699 (3)	0.10082 (19)	0.24040 (15)	0.0213 (5)	
H8	0.6707	0.0870	0.2094	0.026*	
C9	0.7191 (3)	0.14403 (19)	0.31837 (15)	0.0206 (5)	
H9	0.8171	0.1549	0.3508	0.025*	
C10	0.6100 (3)	0.06275 (19)	0.35568 (15)	0.0213 (5)	
H10	0.5151	0.0491	0.3218	0.026*	
C11	0.7058 (3)	−0.03748 (19)	0.36589 (15)	0.0218 (5)	
H11	0.8047	−0.0211	0.3964	0.026*	
C12	0.6157 (4)	−0.1242 (2)	0.40603 (16)	0.0248 (6)	
H121	0.6855	−0.1866	0.4092	0.030*	
H122	0.5888	−0.1022	0.4587	0.030*	
O71	0.9959 (3)	0.01943 (18)	0.29538 (14)	0.0217 (5)	0.77
H71O	1.0752	−0.0218	0.2860	0.026*	0.77
O72	0.9226 (12)	−0.0517 (7)	0.1856 (5)	0.031 (2)	0.23
H72O	0.8861	−0.0216	0.1467	0.037*	0.23
O10	0.5548 (3)	0.09459 (15)	0.42885 (11)	0.0251 (5)	
H10O	0.4729	0.1468	0.4241	0.030*	
O11	0.7561 (2)	−0.07395 (14)	0.29198 (11)	0.0229 (4)	
O12	0.4722 (3)	−0.1495 (2)	0.36648 (17)	0.0485 (7)	
H12O	0.4325	−0.2091	0.3760	0.058*	
N1	0.8734 (3)	0.17181 (16)	0.19831 (13)	0.0214 (5)	
H1N	0.9621	0.1942	0.2205	0.026*	
C13	0.8410 (3)	0.20474 (19)	0.12798 (16)	0.0220 (6)	
O13	0.7167 (3)	0.18034 (14)	0.09211 (11)	0.0258 (4)	
C14	0.9628 (4)	0.2771 (2)	0.09275 (17)	0.0312 (7)	
H141	1.0519	0.2884	0.1287	0.047*	
H142	0.9112	0.3438	0.0810	0.047*	
H143	1.0048	0.2461	0.0456	0.047*	
O1W	0.2143 (3)	0.39008 (16)	0.43738 (13)	0.0370 (6)	
H11W	0.1688	0.3974	0.4806	0.044*	
H12W	0.2950	0.4330	0.4398	0.044*	
O2W	0.8087 (2)	0.72473 (14)	0.24058 (11)	0.0247 (4)	
H21W	0.7630	0.7867	0.2498	0.030*	
H22W	0.7901	0.7083	0.1942	0.030*	

### Atomic displacement parameters (Å^2^)

**Table:**

	*U*^11^	*U*^22^	*U*^33^	*U*^12^	*U*^13^	*U*^23^
C1	0.0228 (13)	0.0155 (11)	0.0234 (12)	−0.0008 (11)	0.0008 (11)	−0.0003 (9)
C2	0.0226 (13)	0.0174 (12)	0.0234 (12)	−0.0030 (10)	0.0052 (11)	0.0017 (10)
C3	0.0216 (12)	0.0146 (11)	0.0284 (13)	−0.0008 (10)	0.0015 (11)	0.0010 (10)
C4	0.0235 (13)	0.0161 (11)	0.0282 (13)	0.0002 (11)	0.0020 (11)	0.0000 (10)
C5	0.0270 (13)	0.0168 (11)	0.0266 (13)	−0.0004 (11)	−0.0002 (11)	0.0000 (10)
C6	0.0334 (16)	0.0232 (13)	0.0271 (14)	0.0061 (12)	0.0018 (12)	0.0011 (11)
O1	0.0240 (10)	0.0130 (8)	0.0280 (9)	0.0006 (8)	−0.0020 (8)	−0.0001 (7)
O2	0.0269 (10)	0.0221 (9)	0.0233 (9)	−0.0041 (8)	0.0020 (8)	0.0008 (7)
O3	0.0302 (11)	0.0151 (8)	0.0329 (10)	0.0033 (8)	0.0029 (9)	0.0043 (7)
O4	0.0272 (10)	0.0196 (9)	0.0336 (10)	0.0058 (8)	0.0065 (9)	0.0026 (8)
O5	0.0286 (10)	0.0147 (8)	0.0225 (9)	0.0002 (8)	0.0013 (8)	−0.0005 (7)
O6	0.0311 (11)	0.0193 (9)	0.0293 (10)	0.0021 (8)	−0.0036 (8)	0.0022 (8)
C7	0.0244 (13)	0.0165 (11)	0.0251 (12)	−0.0016 (11)	0.0001 (11)	0.0006 (10)
C8	0.0255 (13)	0.0146 (11)	0.0237 (12)	−0.0025 (11)	0.0002 (11)	0.0029 (10)
C9	0.0212 (12)	0.0138 (11)	0.0268 (12)	0.0010 (11)	−0.0009 (11)	0.0000 (10)
C10	0.0254 (14)	0.0160 (11)	0.0225 (12)	0.0003 (11)	0.0018 (11)	−0.0005 (10)
C11	0.0252 (13)	0.0162 (11)	0.0241 (12)	0.0004 (11)	0.0004 (11)	−0.0004 (10)
C12	0.0257 (14)	0.0182 (12)	0.0304 (13)	0.0001 (11)	−0.0022 (12)	0.0039 (11)
O71	0.0196 (11)	0.0169 (11)	0.0284 (12)	0.0029 (10)	−0.0022 (10)	−0.0015 (9)
O72	0.039 (5)	0.024 (4)	0.028 (4)	0.008 (4)	0.011 (4)	0.001 (4)
O10	0.0307 (11)	0.0200 (9)	0.0246 (9)	0.0042 (8)	0.0048 (8)	0.0018 (8)
O11	0.0289 (10)	0.0153 (8)	0.0246 (9)	−0.0021 (8)	0.0027 (8)	−0.0012 (7)
O12	0.0368 (13)	0.0384 (12)	0.0703 (17)	−0.0204 (11)	−0.0240 (13)	0.0300 (12)
N1	0.0208 (10)	0.0168 (10)	0.0268 (11)	−0.0020 (9)	0.0010 (9)	0.0007 (9)
C13	0.0235 (13)	0.0148 (11)	0.0278 (12)	−0.0005 (10)	−0.0005 (11)	0.0002 (10)
O13	0.0256 (10)	0.0232 (9)	0.0286 (10)	−0.0046 (8)	−0.0015 (8)	0.0015 (8)
C14	0.0330 (16)	0.0308 (14)	0.0298 (14)	−0.0107 (13)	0.0024 (12)	0.0058 (12)
O1W	0.0484 (14)	0.0261 (10)	0.0365 (12)	−0.0066 (11)	0.0090 (11)	−0.0014 (9)
O2W	0.0292 (10)	0.0169 (8)	0.0280 (9)	0.0031 (8)	−0.0015 (9)	−0.0020 (7)

### Geometric parameters (Å, °)

**Table:**

C1—O1	1.394 (3)	C8—N1	1.452 (3)
C1—O5	1.426 (3)	C8—C9	1.532 (4)
C1—C2	1.525 (3)	C8—H8	1.0000
C1—H1	1.0000	C9—C10	1.528 (4)
C2—O2	1.419 (3)	C9—H9	1.0000
C2—C3	1.532 (3)	C10—O10	1.420 (3)
C2—H2	1.0000	C10—C11	1.523 (3)
C3—O3	1.414 (3)	C10—H10	1.0000
C3—C4	1.518 (4)	C11—O11	1.437 (3)
C3—H3	1.0000	C11—C12	1.514 (4)
C4—O4	1.429 (3)	C11—H11	1.0000
C4—C5	1.523 (3)	C12—O12	1.413 (4)
C4—H4	1.0000	C12—H121	0.9900
C5—O5	1.432 (3)	C12—H122	0.9900
C5—C6	1.516 (4)	O71—H71O	0.8594
C5—H5	1.0000	O72—H72O	0.8400
C6—O6	1.425 (3)	O10—H10O	0.9571
C6—H61	0.9900	O12—H12O	0.8497
C6—H62	0.9900	N1—C13	1.329 (4)
O1—C9	1.434 (3)	N1—H1N	0.8800
O2—H2O	0.8500	C13—O13	1.246 (4)
O3—H3O	0.8500	C13—C14	1.504 (4)
O4—H4O	0.9338	C14—H141	0.9800
O6—H6O	0.8499	C14—H142	0.9800
C7—O71	1.380 (4)	C14—H143	0.9800
C7—O11	1.428 (3)	O1W—H11W	0.8500
C7—O72	1.435 (9)	O1W—H12W	0.8676
C7—C8	1.538 (3)	O2W—H21W	0.8963
C7—H71	1.0000	O2W—H22W	0.8523
C7—H72	1.0000		
			
O1—C1—O5	108.1 (2)	O72—C7—H72	107.2
O1—C1—C2	108.3 (2)	C8—C7—H72	107.4
O5—C1—C2	110.2 (2)	N1—C8—C9	112.7 (2)
O1—C1—H1	110.1	N1—C8—C7	109.2 (2)
O5—C1—H1	110.1	C9—C8—C7	108.5 (2)
C2—C1—H1	110.1	N1—C8—H8	108.8
O2—C2—C1	110.1 (2)	C9—C8—H8	108.8
O2—C2—C3	107.2 (2)	C7—C8—H8	108.8
C1—C2—C3	111.0 (2)	O1—C9—C10	110.9 (2)
O2—C2—H2	109.5	O1—C9—C8	110.3 (2)
C1—C2—H2	109.5	C10—C9—C8	107.2 (2)
C3—C2—H2	109.5	O1—C9—H9	109.5
O3—C3—C4	107.5 (2)	C10—C9—H9	109.5
O3—C3—C2	111.3 (2)	C8—C9—H9	109.5
C4—C3—C2	112.4 (2)	O10—C10—C11	107.8 (2)
O3—C3—H3	108.5	O10—C10—C9	112.3 (2)
C4—C3—H3	108.5	C11—C10—C9	108.6 (2)
C2—C3—H3	108.5	O10—C10—H10	109.4
O4—C4—C3	111.0 (2)	C11—C10—H10	109.4
O4—C4—C5	109.4 (2)	C9—C10—H10	109.4
C3—C4—C5	109.9 (2)	O11—C11—C12	108.7 (2)
O4—C4—H4	108.8	O11—C11—C10	108.7 (2)
C3—C4—H4	108.8	C12—C11—C10	114.8 (2)
C5—C4—H4	108.8	O11—C11—H11	108.2
O5—C5—C6	107.9 (2)	C12—C11—H11	108.2
O5—C5—C4	110.9 (2)	C10—C11—H11	108.2
C6—C5—C4	112.3 (2)	O12—C12—C11	110.9 (2)
O5—C5—H5	108.5	O12—C12—H121	109.5
C6—C5—H5	108.5	C11—C12—H121	109.5
C4—C5—H5	108.5	O12—C12—H122	109.5
O6—C6—C5	112.3 (2)	C11—C12—H122	109.5
O6—C6—H61	109.2	H121—C12—H122	108.0
C5—C6—H61	109.2	C7—O71—H71O	113.3
O6—C6—H62	109.2	C7—O72—H72O	109.5
C5—C6—H62	109.2	C10—O10—H10O	110.6
H61—C6—H62	107.9	C7—O11—C11	113.28 (19)
C1—O1—C9	115.2 (2)	C12—O12—H12O	115.9
C2—O2—H2O	114.2	C13—N1—C8	123.4 (2)
C3—O3—H3O	110.2	C13—N1—H1N	118.3
C4—O4—H4O	105.5	C8—N1—H1N	118.3
C1—O5—C5	111.8 (2)	O13—C13—N1	123.6 (3)
C6—O6—H6O	113.0	O13—C13—C14	120.2 (2)
O71—C7—O11	111.5 (2)	N1—C13—C14	116.2 (2)
O11—C7—O72	109.2 (4)	C13—C14—H141	109.5
O71—C7—C8	107.1 (2)	C13—C14—H142	109.5
O11—C7—C8	109.4 (2)	H141—C14—H142	109.5
O72—C7—C8	115.9 (4)	C13—C14—H143	109.5
O71—C7—H71	109.6	H141—C14—H143	109.5
O11—C7—H71	109.6	H142—C14—H143	109.5
C8—C7—H71	109.6	H11W—O1W—H12W	103.1
O11—C7—H72	107.4	H21W—O2W—H22W	108.4
			
O1—C1—C2—O2	−69.5 (3)	O11—C7—C8—C9	58.8 (3)
O5—C1—C2—O2	172.4 (2)	O72—C7—C8—C9	−177.2 (5)
O1—C1—C2—C3	171.9 (2)	C1—O1—C9—C10	118.0 (2)
O5—C1—C2—C3	53.9 (3)	C1—O1—C9—C8	−123.3 (2)
O2—C2—C3—O3	70.7 (3)	N1—C8—C9—O1	58.5 (3)
C1—C2—C3—O3	−169.0 (2)	C7—C8—C9—O1	179.6 (2)
O2—C2—C3—C4	−168.6 (2)	N1—C8—C9—C10	179.3 (2)
C1—C2—C3—C4	−48.3 (3)	C7—C8—C9—C10	−59.5 (3)
O3—C3—C4—O4	50.3 (3)	O1—C9—C10—O10	−59.4 (3)
C2—C3—C4—O4	−72.6 (3)	C8—C9—C10—O10	−179.9 (2)
O3—C3—C4—C5	171.5 (2)	O1—C9—C10—C11	−178.5 (2)
C2—C3—C4—C5	48.6 (3)	C8—C9—C10—C11	61.0 (3)
O4—C4—C5—O5	66.6 (3)	O10—C10—C11—O11	177.2 (2)
C3—C4—C5—O5	−55.5 (3)	C9—C10—C11—O11	−60.9 (3)
O4—C4—C5—C6	−54.2 (3)	O10—C10—C11—C12	55.2 (3)
C3—C4—C5—C6	−176.4 (2)	C9—C10—C11—C12	177.1 (2)
O5—C5—C6—O6	79.5 (3)	O11—C11—C12—O12	−64.1 (3)
C4—C5—C6—O6	−157.9 (2)	C10—C11—C12—O12	57.9 (3)
O5—C1—O1—C9	−81.6 (3)	O71—C7—O11—C11	57.6 (3)
C2—C1—O1—C9	159.0 (2)	O72—C7—O11—C11	171.4 (5)
O1—C1—O5—C5	179.3 (2)	C8—C7—O11—C11	−60.7 (3)
C2—C1—O5—C5	−62.6 (3)	C12—C11—O11—C7	−172.7 (2)
C6—C5—O5—C1	−172.6 (2)	C10—C11—O11—C7	61.8 (3)
C4—C5—O5—C1	64.0 (3)	C9—C8—N1—C13	−125.0 (3)
O71—C7—C8—N1	61.1 (3)	C7—C8—N1—C13	114.2 (3)
O11—C7—C8—N1	−178.0 (2)	C8—N1—C13—O13	2.1 (4)
O72—C7—C8—N1	−53.9 (5)	C8—N1—C13—C14	−179.2 (2)
O71—C7—C8—C9	−62.2 (3)		

### Hydrogen-bond geometry (Å, °)

**Table:**

*D*—H···*A*	*D*—H	H···*A*	*D*···*A*	*D*—H···*A*
N1—H1N···O2W^i^	0.88	2.05	2.923 (3)	169
O2—H2O···O12^ii^	0.85	1.80	2.642 (3)	170
O3—H3O···O2W	0.85	1.86	2.702 (3)	169
O4—H4O···O13^ii^	0.93	1.95	2.803 (3)	150
O6—H6O···O1W^iii^	0.85	1.79	2.624 (3)	168
O10—H10O···O6^iv^	0.96	1.81	2.705 (3)	154
O1W—H11W···O10^iv^	0.85	1.85	2.696 (3)	175
O12—H12O···O13^v^	0.85	1.96	2.784 (3)	162
O1W—H12W···O4	0.87	1.90	2.759 (3)	173
O2W—H21W···O11^vi^	0.90	1.94	2.772 (3)	154
O2W—H22W···O6^vii^	0.85	1.91	2.757 (3)	171
O71—H71O···O2^i^	0.86	1.87	2.683 (3)	159
O72—H72O···O1W^v^	0.84	2.04	2.545 (9)	119

Symmetry codes: (i) −*x*+2, *y*−1/2, −*z*+1/2; (ii) −*x*+1, *y*+1/2, −*z*+1/2; (iii) *x*+1/2, −*y*+1/2, −*z*+1; (iv) *x*−1/2, −*y*+1/2, −*z*+1; (v) −*x*+1, *y*−1/2, −*z*+1/2; (vi) *x*, *y*+1, *z*; (vii) −*x*+3/2, −*y*+1, *z*−1/2.
